# Role of microRNA-130a in the pathogeneses of obstructive sleep apnea hypopnea syndromeassociated pulmonary hypertension by targeting the GAX gene: Retraction

**DOI:** 10.1097/MD.0000000000024258

**Published:** 2021-01-08

The article, “Role of microRNA-130a in the pathogeneses of obstructive sleep apnea hypopnea syndromeassociated pulmonary hypertension by targeting the GAX gene”,^[1]^ which appeared in Volume 96, Issue 20 of *Medicine*, is being retracted by request of the authors due to sampling labelling errors. The authors reported that the cell grouping markers for the miR-130a mimic group and miR-130a inhibitor group were reversed, resulting in an incorrect conclusion. The operational error led to opposite experimental results in repeated Western blotting experiments in a later stage and incorrect experimental data.
